# The Roles of Phosphorus and Nitrogen Nutrient Transporters in the Arbuscular Mycorrhizal Symbiosis

**DOI:** 10.3390/ijms231911027

**Published:** 2022-09-20

**Authors:** Wenjing Rui, Zhipeng Mao, Zhifang Li

**Affiliations:** Beijing Key Laboratory of Growth and Developmental Regulation for Protected Vegetable Crops, Department of Vegetable Science, College of Horticulture, China Agricultural University (CAU), Haidian District, Yuanmingyuanxilu 2, Beijing 100193, China

**Keywords:** arbuscular mycorrhizal, phosphorus, nitrogen, transporters

## Abstract

More than 80% of land plant species can form symbioses with arbuscular mycorrhizal (AM) fungi, and nutrient transfer to plants is largely mediated through this partnership. Over the last few years, great progress has been made in deciphering the molecular mechanisms underlying the AM-mediated modulation of nutrient uptake progress, and a growing number of fungal and plant genes responsible for the uptake of nutrients from soil or transfer across the fungal–root interface have been identified. In this review, we outline the current concepts of nutrient exchanges within this symbiosis (mechanisms and regulation) and focus on P and N transfer from the fungal partner to the host plant, with a highlight on a possible interplay between P and N nutrient exchanges. Transporters belonging to the plant or AM fungi can synergistically process the transmembrane transport of soil nutrients to the symbiotic interface for further plant acquisition. Although much progress has been made to elucidate the complex mechanism for the integrated roles of nutrient transfers in AM symbiosis, questions still remain to be answered; for example, P and N transporters are less studied in different species of AM fungi; the involvement of AM fungi in plant N uptake is not as clearly defined as that of P; coordinated utilization of N and P is unknown; transporters of cultivated plants inoculated with AM fungi and transcriptomic and metabolomic networks at both the soil–fungi interface and fungi–plant interface have been insufficiently studied. These findings open new perspectives for fundamental research and application of AM fungi in agriculture.

## 1. Introduction

Phosphorus (P) and nitrogen (N) are key nutrients that play major roles in crop production. However, there is not enough P in the soil that can be directly used by plants. On the one hand, most soil P is bound to organic molecules or mineral surfaces, or precipitated as insoluble phosphate (Pi); on the other hand, the mobility of P in soil is low, so it is more difficult for plants to obtain P [1]. In many ecosystems, Pi levels limit plant growth, which has a significant impact on agriculture, especially in areas where low-input agriculture is practiced. In addition, nitrate (NO_3_^−^) and ammonium (NH_4_^+^) and organic N in the forms of amino acids and peptides are the dominant forms of N that are available to plants, and plant N use efficiency is low, rarely exceeding 50% in most agricultural systems due to leaching and run-off losses and gaseous N emissions [2,3]. Because of the status of Pi and N in soil, there is a great demand for chemical fertilizers to improve nutrient deficiency [4]. According to the International Fertilizer Association (IFA) (https://www.fertilizer.org/ accessed on 1 September 2021, by the end of 2020, the global requirement of chemical fertilizers of N, P, and K was expected to reach 260 million tons. Excessive application of chemical fertilizer will not only increase the production cost but also cause a series of environmental problems. Plants have evolved a number of physiological changes to overcome scarce levels of Pi and N, and one of these strategies is to form symbiotic associations with AM fungi [5].

Over 80% of terrestrial plant species can form symbiotic relationships with AM fungi, and these partnerships began more than 450 million years ago [6]. Arbuscules are formed in cortical cells after plant roots were colonized by AM fungi, which are surrounded by a plant-derived periarbuscular membrane (PAM). The PAM and arbuscular membrane form the main symbiotic interface for bidirectional transfers of nutrients between plants and fungi [7]. The fungi facilitate plant uptake of mineral nutrients and the plants supply carbohydrates and lipids to fungi as a source of carbon for their metabolic needs [8]. AM fungi play important roles in terrestrial ecosystems and contribute to improving agricultural productivity; therefore, it is important to understand the regulation of nutrient transport and metabolism associated with the symbiotic state of AM fungi [9]. Previous studies showed that mycorrhizal rice could receive more than 40% of its N, and that 70% of the total P acquired by mycorrhizal rice was through the mycorrhizal pathway [10,11].

In the past, there has been an emphasis on the study of nutrient transport by AM symbiosis from a physiological perspective; however, research indicates an increase in the number of genes that may be involved in the transport of nutrients through the soil fungus interface or the fungus root interface [12]. In this review, we outline the current concepts (mechanisms and regulation) of nutrient exchange in this symbiotic relationship, with a focus on P and N transfer from the fungal partner to the host plant.

## 2. Development of AM Symbiosis

**Figure 1:**
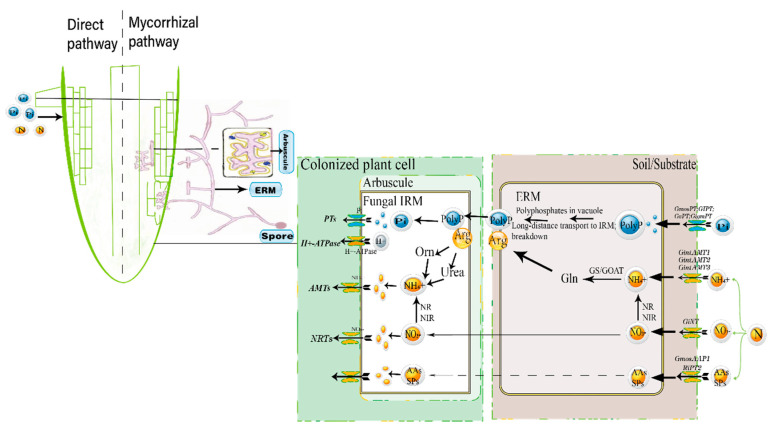
Diagrammatic illustration of the two nutrient uptake pathways in AM plants. Notes: Mechanisms of nutrient acquisition in plants. All arbuscular mycorrhizal (AM) host plants are capable of direct and indirect uptake of phosphate and nitrogen. In the mycorrhiza pathway, the plants absorb soil nutrients via Pi transporters (PTs), NH_4_^+^ transporters (AMTs), and NO_3_^−^ transporters (NRTs). The NO_3_^−^ and NH_4_^+^ are metabolized into arginine (Arg) and transported to the intraradical mycelium (IRM) in cooperation with the polyphosphates (PolyP) driven from Pi. The PolyP–Arg complex is then hydrolyzed to NH_4_^+^ and Pi in the intraradical mycelium (IRM) and transmembrane transported into the plant via the PTs, NRTs, and AMTs at the fungal–plant plasma membranes.

The history of mycorrhizae started with their description, interpretation, and naming by A.B. Frank in 1885 [13]. The formation of them is the result of long-term coevolution between the root system of plants and AM fungi, AM association requires the creation of a novel symbiotic interface within the root cells [2]. Development of AM symbiosis starts with a signal exchange between AM fungi and host plants [14], which can be divided into distinct stages: (1) At the onset of symbiosis, plants secrete strigolactones (SLs) to stimulate spore germination and hyphal branching near the root [15]. SLs, a class of carotenoid-derived terpenoid lactones, were initially characterized as germination stimulants for root parasitic plants such as witchweeds. After 40 years, SLs were identified to be root-derived symbiotic signals for AM fungi [16], this is also a very important factor that triggered the change in the development of AM fungi. (2) Once fungi perceive SLs, AM fungi release signaling molecules termed “mycorrhizal factors”, including lipochitooligosaccharides (LCOs) and short chitin oligomers (Cos), to activate a common symbiotic signaling pathway [17]. (3) Then, fungal hyphae penetrate the root epidermis, invade cortical cells, and develop highly branched tree-like structures called arbuscules, where the nutrient exchange between fungus and plant takes place. Mycorrhizal colonized roots have two pathways for nutrient absorption: direct absorption through root epidermis or root hairs (direct pathway), and indirect absorption via AM fungi hyphae (mycorrhizal pathway) [18,19] (Figure 1). A recent study by Shi et al. [20] demonstrated that P starvation response transcription factors connect the mycorrhizal P uptake pathway with the direct P uptake pathway to improve P absorption.

## 3. Mechanisms of P Uptake in Plants

### 3.1. P Uptake System in Plants: Lessons from Arabidopsis and Tomato

In the direct pathway, Pi is acquired mainly through low or high H^+^/Pi transporters on the epidermis. These phosphate transporters (PHTs) are either constitutively expressed or induced to be expressed in response to Pi deficiency. In roots, Pi is taken up through PHTs and loaded into the xylem for translocation to the shoots [21,22,23].

**Figure 2:**
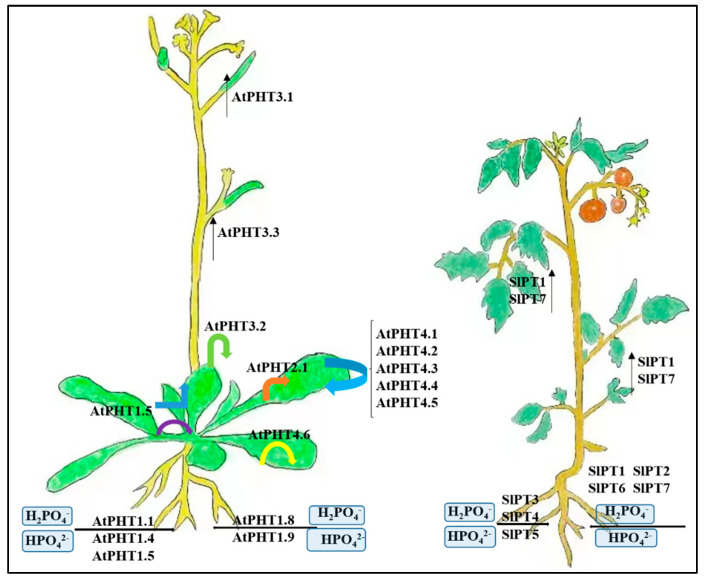
Integrative model to illustrate physiological functions of phosphate transporters in Ara-bidopsis and tomato. Detailed illustration of phosphate uptake and translocation in Arabidopsis and tomato.

In Arabidopsis, PHTs involved in the transfer of Pi into plant cells have been identified, and these proteins have been classified into four families: PHT1, PHT2, PHT3, and PHT4 (Figure 2) [24]. Nine transporters of the PHT1 family have been identified in Arabidopsis: *AtPHT1.1–AtPHT1.9*. The functions of *AtPHT1.1*, *AtPHT1.4*, *AtPHT1.5*, *AtPHT1.8*, and *AtPHT1.9* have been defined, and all of them are high-affinity PHTs that are localized in the plasma membrane and induced by Pi starvation. *AtPHT1.1* and *AtPHT1.4* are the most highly expressed PHTs in Arabidopsis [25]. The polypeptides encoded by *AtPHT1.1* and *AtPHT1.4* showed 78% similarity to each other and showed high amino acid sequence similarity to the high-affinity PHTs of *Saccharomyces cerevisiae*, *Neurospora crassa*, and *Glomus versiforme* [26,27,28]. *AtPHT1.8* and *AtPHT1.9* are highly expressed in roots and are also involved in Pi absorption [29]. In Arabidopsis roots and rosette leaves, Pi deficiency resulted in increased abundance of *AtPHT1.5* transcripts, which are required for proper translocation of *AtPHT1.5* Pi from roots to stems under these conditions. However, the expression of *AtPHT1.5* under Pi replete conditions is different from that under Pi-deficient conditions. *AtPHT1.5* is mainly expressed in shoots and is responsible for Pi transport to sinks and from shoots to roots under Pi-replete conditions [30]. *AtPHT2.1*, the only PHT2 family member in Arabidopsis, is a single-copy gene that shows shoot-specific expression independent of the external Pi concentration, and the localization of the *AtPHT2.1* green fluorescent protein fusion protein indicates that it is present inside the chloroplast intima [31]. In Arabidopsis, PHT3 is also called MPT3, which has three members: *AtMPT1*, *AtMPT2,* and *AtMPT3*. MPTs play a key role in ATP production in plant cells. *AtMPT1* is strongly expressed in the stamens of flowers; *AtMPT2* is highly expressed in senescent leaves; AtMPT3-GUS is strongly expressed in vascular tissues, and the expression of this gene has also been detected in rosette leaves, roots, and meristems of young seedlings. The three *AtMPT* genes have different expression patterns, implying that they play specific roles at different developmental stages [32]. In Arabidopsis, the PHT4 family has six members: five of the proteins are targeted to the plastid envelope, and the sixth resides in the Golgi apparatus, these genes are not responsive to Pi deprivation. The PHT4 gene is expressed in both roots and leaves, with *AtPHT4.1* and *AtPHT4.4* having the highest expression levels in the leaves. With the exception of *AtPHT4.2* and *AtPHT4.6*, the other four members of this gene family are more abundantly expressed in leaves than in roots. Thus, the plastid-localized protein members of this family may function in both heterotrophic plastids and photosynthesis [33].

The PHT1 family in tomato consists of seven members: *SlPT1*–*SlPT7* (Figure 2) [34]. Transcripts of *SlPT1* and *SlPT7* are detected in large numbers in roots and leaves and, to a lesser extent, in stems and flowers as well as in fruits. The relatively high transcript levels of *SlPT1* and *SlPT7* in these sink tissues suggest that they may have evolved to meet the requirements for transporting Pi from source to sink [35]. The expression of *SlPT2* and *SlPT6* show relatively distinct tissue-specific profiles, with their transcripts intensively in roots and extremely faintly in some other tissues [36]. The expression patterns of *SlPT3* and *SlPT5* are relatively similar, and both genes are weakly expressed in all tissues. However, the transcript of *SlPT4* is not detectable in all tissues examined [37]. The expression of *SlPT1, SlPT2, SlPT3, SlPT5, SlPT6,* and *SlPT7* are significantly repressed under high-Pi-supply conditions. The expression of these genes is significantly reduced in response to high P availability, which may be partly due to the significant increase in P concentration in tomato plants under these conditions [38].

### 3.2. Transport of P in AM Symbiosis

The transfer of Pi from AM fungi to host plants was revealed by using isotopically labeled substrates in a two-compartment system [39]. In AM-colonized rice, 70% of Pi was transported via the mycorrhizal pathway [10]. In mycorrhizal plants consisting of the rhizosphere and the hyphosphere, AM fungi take up Pi from the soil through Pi transporters located on the plasma membrane of extraradical hyphae. The absorbed Pi is rapidly converted to polyphosphate (poly-P) and isolated in tubular vesicles, where the IRM long-chain polyphosphate is broken down into [10] shorter chains by a vacuolar endopoly-phosphatase. Afterwards, polyphosphatase hydrolyzes the terminal residues of short-chain polyphosphate and releases Pi, which can be transferred to the host plant through the mycorrhizal interface [40,41,42] (Figure 1).

#### 3.2.1. Pi Transport in AM Fungi

Pi is taken up by the mycelium of AM fungi, transferred to intraradicular fungal structures, and released into the periarbuscular space (PAS) containing arbuscule cells, and the transport proteins may be involved in symbiotic transport by the fungi. In particular, AM fungi have high-affinity Pi/H^+^ transporters, which are homologues of the yeast PHO84 Pi transporter and belong to a family of major transporters similar to plant Pi transporters [3,43]. Four Pi transporters of AM fungi have been identified: *Rhizophagus irregularis* (*GiPT*), *Funneliformis mosseae* (*GmosPT*), *Rhizophagus clarus* (*GvPT*), and *Gigaspora margarita* (*GigmPT*) (Table 1). They all share structural and sequential similarities with other plant and fungal high-affinity PTs [28,43,44,45]. *GvPT* and *GiPT* transcripts were detected mainly in extraradical mycelium, indicating their role in acquiring Pi from the soil. *GiPT* expression was correlated with the external Pi concentration and over all Pi content of mycorrhizal roots. *GmosPT* had high sequence similarity (73% identity and 86% similarity with *GiPT*), and they were expressed in both extra- and intraradical mycelium, but not in germinating and dormant sporocarps. The phylogenetic analysis showed that *GmosPT*, *GvPT,* and *GiPT* groups have close sequences [43,44,46]. In addition, *GigmPT* is considered to be the major Pi sensor in *Gigaspora margarita* and is involved in the acquisition of Pi from PAS via upregulation of the phosphate signaling (PHO) pathway, as well as sensing changes in extracellular Pi through activation of the protein kinase A (PKA) signaling cascade [47].

#### 3.2.2. AM Symbiosis Affects Plant Pi Transport

The Pi uptake pathway of mycorrhizae may dominate Pi uptake in AM symbiosis, which is heavily dependent on AM-induced PHT1 members [48,49]. The transcript levels of many PHT1 transporter proteins decrease with increasing Pi levels, and the expression of a small subgroup of PHT1 transporter proteins in AM symbiosis is actually induced in mycorrhizal roots [3]. In AM symbiosis, two classes are known to be involved in Pi transport: mycorrhiza-specific Pi transporters and mycorrhiza-inducible Pi transporters [50].

Mycorrhizal-specific PHT1 members have been identified in many plants, and are expressed in response to symbiotic relationships (Table 1). Immunolocalization and expression studies on *MtPT4* of *Medicago truncatula* showed that this transporter has a subcellular targeting effect on plant PAM. The rice homolog of *MtPT4*, *OsPT11*, showed a similar localization [51,52,53]. In addition, tracer studies confirmed that Pi was not delivered through the fungus during *os-pt11* symbiosis [10]. Due to gene duplication, there are two orthologues of *OsPT11* in tomato: *SlPT4* and *SlPT5. SlPT4* is exclusively expressed during symbiosis, unlike *OsPT11*, and it is dispensable for symbiotic P uptake.

Phylogenetic tree analysis showed that *SlPT4* and *StPT4* clustered with mycorrhiza-specific Pi transporter proteins from *Medicago truncatula*, indicating that two nonhomologous mycorrhizal response genes encoding Pi transporter proteins are coexpressed in Solanaceae [38]. In addition, extensive sequencing of *StPT4*-like clones and subsequent expression analysis in potato and tomato revealed the existence of a close homologous sequence between *StPT4* and *SlPT4*, named *StPT5* and *SlPT5*, respectively, representing a third Pi transport system in Solanaceae species, which is upregulated upon root colonization by AM fungi [34,37,38]. The AM-inducible Pi transporter of *Lotus japonicus*, *LjPT3*, is expressed in arbuscule-containing cells of the inner cortex. It is especially noteworthy that the *LjPT3* gene is not orthologous to *MtPT4*, therefore, it is possible that other AM-enhanced transporters may exist in *Lotus japonicus* [54]. In *Populus trichocarpa*, only *PtPT10* transcripts were highly enriched in mycorrhizal roots, and promoter sequence analysis revealed conserved motifs in *PtPT10* that were similar to other AM-inducible homologues. Mycorrhizal Pi transport in *Astralagus sinicus* was mediated by the specific Pi transporter *AsPT4* but not *AsPT1*, the loss of *AsPT4* function resulted in a block in symbiotic Pi uptake. Knockdown of *AsPT1* also affected the growth of arbuscules, but did not alter Pi transfer in AM symbiosis, indicating compensatory effects between the two transporters [50]. Reverse genetic analysis showed that *AsPT5* not only mediated Pi translocation and remodeled root system architecture, but was also critical for *Astragalus sinicus* arbuscule formation under moderately high Pi concentrations [55]. In addition, *ZmPT6* is a mycorrhiza-specific Pi transporter gene of maize, and a PT6 mutant showed reduced mycorrhiza formation in maize roots [56]. Most interestingly, the AM-inducible transporter *SbPT10* was only detected in roots colonized by AM fungi, not in leaf or stem tissue [57]. *GmPT10* and *GmPT11* obtained from *Glycine max* are induced upon fungal colonization; however, *GmPT7* is only induced in the later stages of symbiosis [58]. In petunia, the expression of *PhPT4* is mycorrhiza-specific, and *PhPT3* and *PhPT5* are mycorrhiza-inducible, both of them were expressed at low levels in nonmycorrhized roots [59]. *VvPT1* and *VvPT2*, identified in the grape genome, code for putative proteins with a high level of similarity with a series of specific PHTs [60].

Mycorrhiza-inducible PHTs were strongly induced by AM symbiosis, but a small amount was expressed in uninoculated roots (Table 1). RNA hybridization assays in tomato using the *StPT3* probe indicated that *SlPT3* may be a homolog of *StPT3* [34,37], and RNA localization and reporter gene expression indicated that *StPT3* is expressed in root sectors where mycorrhizal structures are formed [61]. In solanaceous species, the AM-regulated PHT genes *SmPT3* in eggplant, *CfPT3* in pepper, and *NtPT3* in tobacco have also been identified [62]. The accumulation of mycorrhizal maize *ZmPT1*, *ZmPT3*, *ZmPT4*, and *ZmPT5* transcripts under low-Pi conditions was positively correlated with shoot biomass, which may be due to P accumulation [63,64]. Homologous *PtPT8* is phylogenetically related to the AM-inducible PHT1 subfamily [65]. In *Brachypodium distachyon*, *BdPT7*, the orthologue of *MtPT4*, was highly induced in mycorrhizal roots, and its transcripts accumulated not only in mycorrhizal roots but also in noncolonized roots and leaves of Pi-starved plants. *BdPT3*, *BdPT12,* and *BdPT13* are also induced during AM symbiosis, similar to *OsPT13* [62,66]. In barley seedlings, AM colonization specifically upregulated the expression of *HvPHT1;11*, *HvPHT1;11.2*, *HvPHT1;12,* and *HvPHT1;13.1*/*13.2* [67]. RT-PCR and in situ hybridization showed that the *HvPT8* and *TaPht-myc* transporters had increased expression in roots colonized by AM fungi [68]. The expression of AM-inducible PHT1 genes (*SbPT9*, *SbPT11*, *LuPT5,* and *LuPT8*) in both root and leaf tissues indicates that these transporters not only play a role in mycorrhizal Pi uptake but also in Pi mobilization in leaves. *SbPT8* also induced nonmycorrhizal roots under low-Pi conditions, suggesting a possible change from the direct Pi uptake pathway to the mycorrhizal Pi uptake pathway during the establishment of AM symbiosis [57]. *Lotus japonicus* allows mycorrhizal plants to take up Pi from their fungal partners and regulates morphogenesis in mycorrhizal plants, and *LjPT4* may play an additional role in the root tip when AM fungi are absent [69].

Entry of Pi into plant cells via PTs through the plasma membrane requires protonation and deprotonation of the transporter, accompanied by conformational changes [19]. In recent years, AM-responsive HA genes have been identified in several plants and are considered key genes for the activation and regulation of the symbiotic interface secondary transport system (Table 1). Notably, *MtHA1* from Medicago and *OsHA1* from rice are the only two known HA genes whose expression is exclusively confined to specific root cells containing AM fungal structures [70,71]. In tobacco, two HA genes (PMA2 and PMA4) were found to be induced in cortical cells containing arbuscules of mycorrhizal roots [72]. A homolog of *OsHA1* and *MtHA1*, *SlHA8*, was identified in tomato, and it was strongly and specifically induced to be expressed in roots colonized by the AM fungi. The *SlHA8* promoter is able to drive GUS reporter gene expression in soybean and rice mycorrhizal roots, suggesting that AM-induced H^+^-ATPase gene expression is highly conserved in different mycorrhizal plants [73,74]. AM symbiosis has received increasing attention for its potential exploitation of the nutrients of crop plants, especially in sustainable agriculture. The function of the arbuscular plasma membrane H^+^-ATPase in energizing nutrient transfer may well be employed in crop improvement [71]. These findings offer new insights into the regulatory mechanism of mineral nutrient uptake by host plants from AM fungi.

AM colonization is usually accompanied by reduced expression of other Pi transporters, especially those involved in the direct pathway (Table 1). In rice, the expression of each gene in relation to AM symbiosis was examined. Although the mRNA levels of *OsPT1*, *OsPT2*, *OsPT3*, *OsPT6*, *OsPT9,* and *OsPT10* expressed in roots declined during symbiosis, in Ospt11 Pi transporter mutants, the expression of *OsPT2* and *OsPT6* genes was not reduced, suggesting an elaborate regulation of Pi acquisition via the direct pathway by the mycorrhizal pathway [10,19,75]. This downregulation of Pi transporters has been observed in potato, and the abundance of the transcripts of *StPT1* and *StPT2* was reduced, probably as a result of the plant fungus interaction and/or an improved Pi status of the mycorrhizal roots [61,76]. Therefore, the root epidermis and AM fungi can balance the uptake of Pi.

### 3.3. Summary and Phylogenetic Analysis of the PHTs

**Figure 3:**
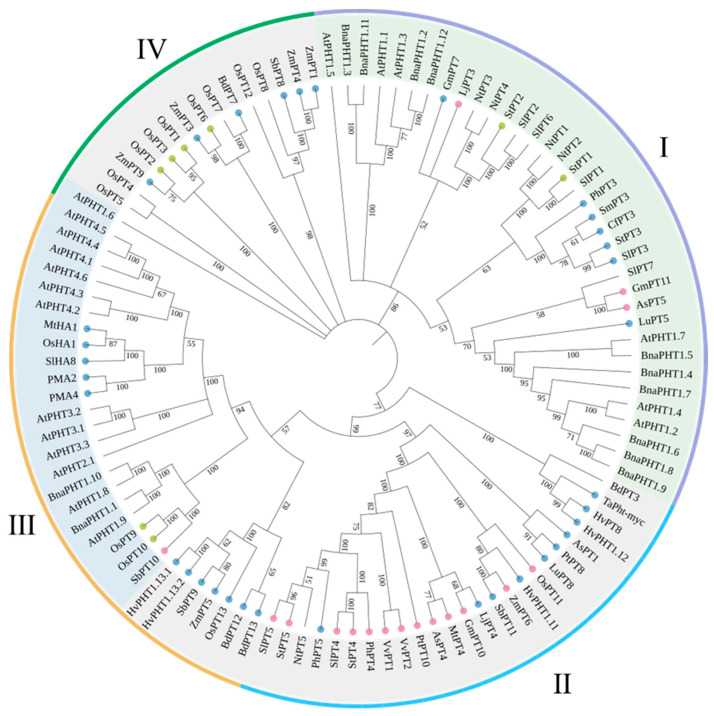
Unrooted dendrogram of plant and fungal Pi transporters. The unrooted phylogenetic tree of the plant phosphate transporters homologs was constructed using their protein sequences by the neighbor joining algorithm within the MEGA7 program with bootstrapping value (range 0 to 100). For tree construction, we used nonmycorrhizal plant *Arabidopsis thaliana* and *Brassica napus* phosphate transporters; mycorrhizal plants *Solanum Lycopersicum*, *Oryza sativa,* and *Nicotiana tabacum* phosphate transporters, and other mycorrhiza-specific, mycorrhizal-induced, and downregulated Pi transporters (Table 1). Mycorrhizal-induced (blue) and downregulated Pi transporters (green) are highlighted in different color.

P is acquired by a mycorrhizal P transporter expressed in ERM and then translocated to IRM, and then taken up by plant cells through PHT proteins. In recent years, there has been a significant increase in our understanding of the physiology and molecular mechanisms of PHT proteins in AM symbiosis. In this part of the review, we discuss the key regulatory role played by PHTs in non-AM (for example, Arabidopsis and tomato) and in AM fungi formation. Sequence comparison between characterized PHTs from the nonmycorrhizal plants *Arabidopsis* and *Brassica napu,* the mycorrhizal plants *Solanum Lycopersicum*, *Oryza sativa*, and *Nicotiana tabacum,* and other AM plant species illustrates some interesting features of the PHT family (Figure 3). The results showed that most mycorrhiza-specific and mycorrhiza-upregulated PHTs clustered into their respective subgroups, and these subgroups contained two mycorrhizal nonresponsive PHTs (*NtPT5* and *BdPT3*) and did not contain any Arabidopsis and *Brassica napus* PHTs. Observations suggest that mycorrhizal plants have PHTs that are adapted to AM symbiosis. On the other hand, the IV subfamily mainly contains mycorrhizae-induced PHTs and four downregulated PHTs. These results indicate that the expression of mycorrhiza-induced PHTs is often accompanied by the downregulation of other PHTs. Interestingly, four mycorrhizae-specific PHT genes (*AsPT5*, *SbPT10*, *GmPT11,* and *LjPT3*) do not cluster with the mycorrhizae-specific subgroup and instead cluster with the other Pht1 members. This may be an evolutionary strategy to ensure a balance between mycorrhizal and direct uptake of P.

We covered the transport proteins involved in Pi fluxes from plants towards fungi, uptake from the soil, and exchange of P together; interestingly, we found P transporters to be less studied in different species of AM fungi. On the plant side, other PHT genes have thus far received less attention than PHT1 family genes in response to mycorrhizal plants. We may pay attention to this research in the future. These advances in the comprehension of PHTs will help underpin the development of crops with optimal P uptake efficiencies.

## 4. Mechanisms of N Uptake in Plants

### 4.1. N Uptake System in Plants: Lessons from Arabidopsis and Tomato 

**Figure 4:**
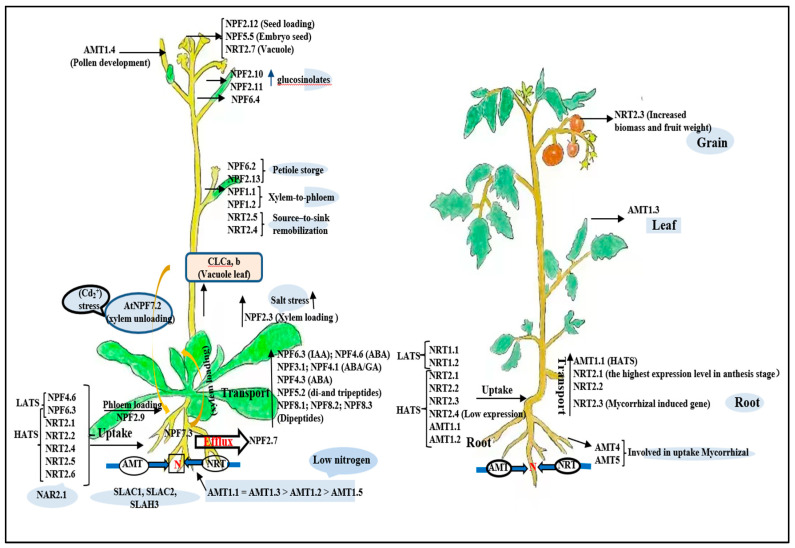
Integrative illustration on physiological functions of those nitrate and ammonium transporters are well investigated using Arabidopsis and tomato as a model. HATS, high-affinity transport system; LATS, low-affinity transport system.

Nitrate and ammonium are the main forms of N in soils [79]. To obtain N from soil, plants have evolved several N-absorbing systems. Understanding the molecular mechanisms of how plants absorb and assimilate N is a critical step to improve plant N use efficiency. To date, five transporter families are known to be involved in N uptake, distribution, or storage: ammonium transporters (AMT), nitrate transporter 1, peptide transporter family (NPF(NRT1/PTR)), nitrate transporter 2 (NRT2), slow anion channel-associated homologues (SLAC/SLAH), and chloride channel family (CLC). In this section, the current studies of N transporters in Arabidopsis and tomato are reviewed (Figure 4).

*AtNPF6.3* (*CHL1*/*AtNRT1.1*) is the first identified nitrate transporter, functioning in nitrate uptake in roots and nitrate translocation from roots to shoots [80,81]. *AtNPF2.7* (*NAXT1*) mainly expresses the cortex of mature roots and is implicated in root nitrate uptake [82]. *AtNPF4.6* (*NRT1.2*/*AIT1*) encodes a constitutive component of the low-affinity nitrate uptake transporter [83]. *AtNPF3.1* (*AtNirt*) encodes a pathogen-induced NO_3_^−^/NO_2_^−^ transporter [84]. *AtNPF7.3* (*NRT1.5*) is a low-affinity, pH-dependent bidirectional nitrate transporter, located in the plasma membrane and expressed in the periplasmic cells of the root whole near the xylem, responsible for xylem nitrate loading [85]. *AtNPF7.2* (*NRT1.8*) is expressed predominantly in xylem parenchyma cells and plays an important role in plant removal of nitrate from xylem vessels [86]. *AtNPF2.9* (*NRT1.9*) may facilitate loading of nitrate into the root phloem and enhance downwards transport of nitrate in roots [87]. *AtNPF2.10* and *AtNPF2.11* are expressed in leaves and silique walls, and regulate the loading of glucosinolates from the apoplasm into the phloem [88]. *AtNPF2.3* is a constitutively expressed transporter whose contribution to NO_3_^−^ translocation to the shoot is quantitatively and physiologically significant under salinity [89]. *AtNPF6.2* (*AtNRT1.4*) regulates nitrate homeostasis in leaves, whose deficiency can alter leaf development [90]. *AtNPF2.13* (*NRT1.7*) is expressed in leaf veinlet siliques and is responsible for silique loading of nitrate in the source leaf to allow nitrate translocation from older to younger leaves [91]. The low-affinity nitrate transporter *AtNPF2.12* (*NRT1.6*) is only expressed in reproductive tissue and is involved in delivering nitrate from maternal tissue to the developing embryo [92]. Another transporter expressed in seeds, *AtNPF5.5,* also affects N accumulation in embryos [93]. *AtNPF1.1* (*NRT1.12*) and *AtNPF1.2* (*NRT1.11*) are low-affinity nitrate transporters that are also involved in transferring nitrate from xylem to phloem [94]. *AtNPF4.1* and *AtNPF4.3* can transport abscisic acid [95]. *AtNPF5.2* protects the plant against biotic and abiotic stresses and transports dipeptides and tripeptides [96]. *AtNPF6.4* is induced by NO_3_^−^ in the leaves, but its expression is inhibited in the roots. *AtNPF8.1, AtNPF8.2,* and *AtNPF8.3* are localized at the plasma membrane, and facilitate the transport of dipeptides with high affinity [95,97]. *SlNRT1.1* and *SlNRT1.2* are the first N transporters identified in tomato that mediate nitrate uptake in roots [98]; they were also found to be low-affinity nitrate transporters, and their expression was root-cell-specific and regulated by N availability. *SlNRT1.1* can improve nitrate uptake in grafted tomato plants under high N demand [99].

In contrast to the transporters of the NRT1 family, the NRT2 family mainly regulates the high-affinity transport system (Figure 4). With the exception of *AtNRT2.7*, all remaining NRT2 transporters interacted strongly with *AtNAR2.1* [100]. Disruption of *AtNRT2.1* and *AtNRT2.2* reduces the inducible high-affinity transport system by up to 80%, and the constitutive high-affinity transport system is reduced by 30% [101]. The expression of *AtNRT2.4* is induced under N starvation, and it is mainly expressed in the epidermis of lateral roots and the phloem of shoots [102]. *AtNRT2.5* is a plasma membrane-localized high-affinity nitrate transporter protein that plays an important role in adult plants under severe N starvation [103]. *AtNRT2.6* is involved in rhizobacterium-stimulated lateral root growth. *SlNRT2.1* and *SlNRT2.2* are highly similar in coding regions, the expression of *SlNRT2.1* and *SlNRT2.2* are restricted to roots, and the highest expression level of *SlNRT2.1* occurs in the anthesis stage [104]. *SlNRT2.3* formation was positively controlled by nitrate and negatively by ammonium, but not by glutamine, and it is expressed in tomato roots colonized by AM fungus [105]. The *SlNRT2.4* gene was expressed in several tissues and organs with the lowest expression level. For subcellular localization, all NRT2 proteins were predicted to be in the plasma membrane [106].

In addition to the NRT1 and NRT2 families, some members of the CLC family also have nitrate transport capacity. In Arabidopsis, CLCa and CLCb act as proton–nitrate exchanges and are more selective for nitrate than for chloride [107].

Previous studies on phylogenetic analyses of the AMT gene family revealed two distinct subfamilies (Figure 4): the AMT1 subfamily (AMT1 cluster) and the AMT2 subfamily (AMT2/3/4 cluster) [108]. The AMT1 cluster gene encodes a protein with high affinity for NH_4_^+^ transport. Transcriptome and RNA gel blot analyses showed that four of the six AMT homologues in Arabidopsis are expressed in roots and upregulated under N-deficient conditions [109]. *AtAMT1.1* is a root NH_4_^+^ transporter that confers approximately one-third of the overall high-affinity transport capacity of N-deficient Arabidopsis [110]. Similar to *AtAMT1.1*, *AtAMT1.3* also accounted for approximately 30% of the overall ammonium uptake capacity in N-deficient Arabidopsis roots. Root ammonium influx in N-deficient plants was 60–70% lower in the *atamt1.1* and *atamt1.3* double mutants, indicating that *AtAMT1.1* and *AtAMT1.3* are functionally additive under N-deficient conditions [111]. However, *AtAMT1.4* is specifically expressed in pollen, which contributes to N nutrients in pollen via NH_4_^+^ uptake or retrieval [108]. *AtAMT1.5* accounts for the remaining ammonium uptake capacity [109]. *AtAMT2* may play a role in the transport of NH_4_^+^ from the apoplast to the symplast [112]. *SlAMT1.1* was the first ammonium transporter identified in tomato to function not only in ammonium uptake but also in ammonium translocation from roots to shoots; it was also found to be strongly induced under low N, and downregulated by drought and salt stress [98,113,114]. *SlAMT1.2* was strongly induced by NH_4_^+^ and NO_3_^−^, increased in leaves at the onset of light, and decreased when CO was elevated. In contrast to other AMT transporters, *SlAMT1.3* is exclusively detected in the leaves and is lower in the light period, higher in the dark, and decreased with elevated CO [113]. Ruzicka et al. [115] identified two novel tomato ammonium transporter genes, *SlAMT4* and *SlAMT5*. Quantitative real-time PCR (qPCR) analysis revealed that they were exclusively expressed in mycorrhizal roots, although they were not significantly regulated by NH_4_^+^ treatments.

### 4.2. N Assimilation in Arabidopsis and Tomato

For many plants, roots take up nitrate and assimilate it, where it is first reduced to nitrate-by-nitrate reductase in the cytoplasm and then further to ammonium by nitrate reductase in the plastids and synthetase (GS) in the plastids and cytoplasm, and then transported to the shoot. The ammonium derived from nitrate or directly ammonium uptake by ammonium transporters (AMTs) is further assimilated into amino acids via the GS/glutamine-2-oxoglutarate aminotransferase (GOGAT) cycle [116]. Plant GS occurs in most species as a single isoform in plastids (GS2) and as three to five isoforms localized in the cytosol (GS1). Cytosolic GS1 is important for primary NH_4_^+^ assimilation in roots and for reassimilation of NH_4_^+^ generated during protein turnover in leaves, whereas the dominating role of GS2 is in reassimilation of photorespiratory NH_4_^+^ in chloroplasts and assimilation of NH_4_^+^ derived from NO_3_^−^ reduction in plastids [117,118]. Fd-GOGAT and NADH-GOGAT are two kinds of GOGAT species from higher plants, while Fd-GOGAT is derived from photorespiration and mainly assimilates ammonium in leaves. NADH-GOGAT is highly expressed in roots [119]. In Arabidopsis, *GLN1;1*, *GLN1;2,* and *GLN1;4* are induced during leaf senescence, and *GLN1;1* is located in the root surface, root tips, and root hairs. *GLN1;2* and *GLN1;3* are localized in the vasculature, and *GLN1;2* is the only one that is significantly upregulated by ammonium. *GLN1;3* is expressed in the root mature zone. *GLN1;4* is expressed within the basal region of lateral root emergence. In Arabidopsis, two *Fd-GOGATs* have been identified: *GLU1* and *GLU2*. *GLU1* is expressed abundantly in the leaves, while *GLU2* is mainly expressed in the roots [79]. In tomato, Liu et al. [120] identified six GS genes (*SlGSI*, *SlGS2,* and *SlGS1.1*–*1.4*) and two GOGAT genes (*SlNADH-GOGAT* and *SlFd-GOGAT*). These genes underwent species-specific evolution, and may have specific biological functions in vivo. *SlGS1.1*, *SlGS1.2*, *SlGS1.3,* and *SlNADH-GOGAT* may cooperatively play significant roles in primary N assimilation in roots.

**Figure 5:**
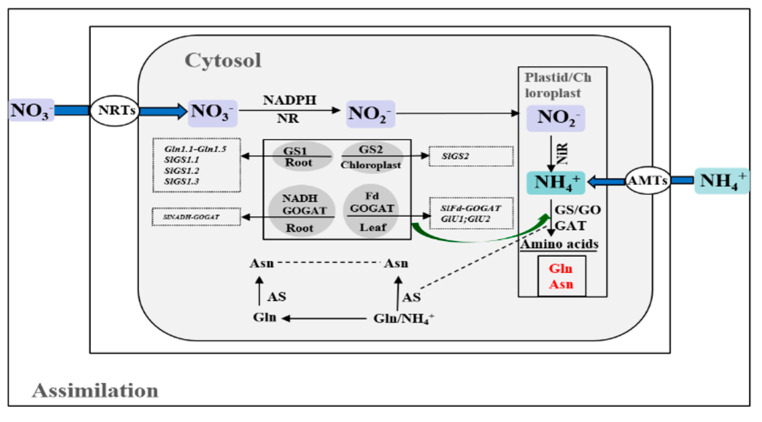
The diagram depicts the main events in the nitrate assimilation lessons from Arabidopsis and tomato.

Three enzymes also probably participate in ammonium assimilation, except for the GS/GOGAT cycle. Cytosolic asparagine synthetase (AS) catalyzes the ATP-dependent transfer of the amido group of glutamines to a molecule of aspartate to generate glutamate and asparagine [121] (Figure 5).

### 4.3. Transport of N in AM Symbiosis

For N, the supply rate of N depends on the mineralization of organic N into inorganic N (NH_4_^+^ or NO_3_^−^) by microorganisms [122]. Determination of the ^15^NO_3_^−^ and ^15^NH_4_^+^ uptake rates indicated that AM fungi contributed more than the N uptake of the plants alone [123]. AM fungi maintains an extraradical mycelium that can extend several centimeters from the root. The IRM within the root are connected to the ERM and form a single continuum [124]. Many studies have reported that ERM can take up 42% of N via the mycorrhizal uptake pathway to plants [125,126]. Seventy five percent of the of N found in *Zea maize* leaves was taken up via the ERM of *Glomus aggregatum* [127]. According to current knowledge, AM fungi take up of N in the ERM is assimilated through the GS/GOGAT cycle, preferentially in the form of ammonium, and metabolizes it to arginine, which is the main form in which N is transported from the ERM to the intraradical mycelium (IRM) and broken down into urea and ornithine in the IRM. Ammonium, the product of urea hydrolyzation, is subsequently released to the symbiotic interface and taken up by plants [128,129,130].

#### 4.3.1. N transport System in AM Fungi

AM fungi prefer the direct uptake of NH_4_^+^ owing to the extra energy needed for the reduction of NO_3_^−^, which is required for nitrogen to incorporate into organic compounds [12]. Here, we report three fungal AMT genes that are attained for NH_4_^+^ uptake by AM fungi (Figure 1). The first AMT of AM fungi, *GintAMT1,* characterized from *R. irregularis*, expressed in the ERM, encodes a high-affinity NH_4_^+^ transporter [131]. Functional complementation in an AMT-defective yeast mutant showed that *GintAMT2* encodes a functional NH_4_^+^ transporter, and plasma membrane localization was revealed by polyclonal antibodies against *GintAMT2*. *GintAMT1* and *GintAMT2* are both expressed in ERM and IRM, which participate in NH_4_^+^ uptake in soil solutions and may be involved in the recovery of NH_4_^+^ leaked during fungal metabolism at the symbiotic interface [129]. *GintAMT3* localizes to the fungal membrane and encodes a functional low-affinity transporter [125].

Nitrate uptake by the ERM of *R. irregularis* is probably coupled to a H^+^-symport mechanism [132]. Nitrate transporters have been shown to play vital roles in NO_3_^−^ transport to the ERM. *GiNT*, identified from *R. irregularis*, was shown to be expressed in all AM fungi tissues (spores, arbuscules, ERM, and IRM) and could play an important role in establishing competition for NO_3_^−^ between the plant and AM fungi at the symbiotic interface by regulating bidirectional fluxes (Figure 1) [128,133].

AM fungi can also enhance the decomposition of N capture from complex organic material in soil, and hyphal growth of the fungal partner was increased in the presence of the organic material, independent of the host plant [134]. Amino acid transport systems have been studied extensively in higher plants, yeast, and filamentous fungi. Based on bioinformatics tools, polymerase chain reaction and heterologous expression systems have been used to characterize the *Funneliformis mosseae* amino acid permease (*GmosAAP1*) sequence. *GmosAAP1* was expressed in ERM but not in IRM structures of plants treated with millimolar nitrate concentrations [135]. Genome-wide transcriptomic data obtained from *R. irregularis* were exploited, and *RiPTR2* showed amino acid sequence and transmembrane domain profiles similar to those of members of the PTR2 family of fungal oligopeptide transporters. The *RiPTR2* sequence was able to complement the growth defects of yeast mutants defective in the two well-studied dipeptide transporters. At least in the heterologous system, *RiPTR2* was able to transport Ala-Leu, Ala-Tyr, Tyr-Ala, and other dipeptides [136].

#### 4.3.2. N Transport Systems on the Plant Side

Plant transporters located in PAM are responsible for the capture of nutrients from the periarbuscular apoplast and their delivery into the cytoplasm of cortical cells [12] (Table 2). *LjAMT2.2* is the first plant AMT gene characterized to be involved in N uptake during AM symbiosis, and has been shown to be the highest upregulated gene in a transcriptomic analysis of *Lotus japonicus* roots upon colonization with *Gigaspora margarita* [137]. In tomato, the mycorrhizal specific transporter *SlAMT4* was 68% identical to *LjAMT2.2* [137], and *SlAMT5* shares 64.4% amino acid identity with *SlAMT4* [115]; RT-PCR indicated that five soybean genes (*GmAMT1.4*, *GmAMT3.1*, *GmAMT4.1, GmAMT4.3,* and *GmAMT4.4*) were upregulated in root colonization with AM, and promoter reporter analysis indicated that the most abundantly transcribed gene, *GmAMT4.1*, showed specific expression in arbuscule cortical cells [138]. In poplar, *PtAMT1.1* and *PtAMT1.2* were mycorrhiza-inducible in AM roots [139]. In Sorghum, the relative gene expression of *SbAMT3;1* and *SbAMT4* were significantly (70 and 20 times, respectively) higher in roots colonized by AM fungi than in nonmycorrhized roots, and *SbAMT3;1* and *SbAMT4* genes are expressed in root cortical cells, which makes them ready to accommodate arbuscules [140]. In *M. truncatula*, *MtAMT2;3*, *MtAMT2;4,* and *MtAMT2;5* transcripts increase significantly during AM symbiosis [141].

Aquaporin-mediated membrane transport of ammonia has already been well analyzed, and aquaporins could be a component of the low-affinity ammonia transport system. An AM-specific Nod 26-like intrinsic protein, *MtNIP1* (Table 2), showed strong induction expression during mycorrhization and possibly facilitated the cellular uptake of ammonia [142]. Laser microdissection revealed that the second NIP gene was expressed in the surrounding hyphae-containing cortical cells as well, and the two NIPs might be involved in the uptake of N into host cells [143].

AM fungi can influence NO_3_^−^ uptake of plants by regulating the transcript levels of NRTs (Table 2). AM-induced NO_3_^−^ transporters have been identified in a variety of plant species, including *Medicago truncatula* [6], *Lotus japonicus* [137], and grapevine [144]. When the roots were colonized by AM fungi, the expression of *SlNRT2.3* extended to the inner cortical cells and the transcript levels of *SlNRT2.3* in AM-colonized roots were higher than in noncolonized controls, which may mediate the positive effects of AM fungi on NO_3_^−^ uptake from soil and NO_3_^−^ distribution to the host [105]. Drechsler et al. [145] investigated the transcriptional regulation of 82 rice NPF genes in response to colonization by the AM fungi *R. irregularis* in roots of plants grown under five different nutrition regimes, and the expression of the *NPF6.4*, *NPF2.2,* and *NPF1.3* genes was strongly induced in mycorrhizal roots and depended on the composition of the fertilizer solution. Wang et al. [11] proposed that *OsNPF4.5* is a low-affinity NO_3_^−^ transporter, and mycorrhizal colonization strongly induced the expression of *OsNPF4.5* in rice roots, which is exclusively expressed in the cells containing arbuscules. The orthologues of NPF4.5 in maize (*ZmNPF4.5*) and sorghum (*SbNPF4.5*) were also found to be strongly upregulated in AM fungal-colonized roots [2]. However, the roles of these AM-induced NRTs in symbiotic NO_3_^−^ transfer are still far from well understood due to the lack of precise identification of their subcellular localization and transport activities.

Members of the plant NPF can transport not only NO_3_^−^ but also oligopeptides across the plasma membrane [95]. Guether et al. [146] demonstrated that peptide transporter (*LjPTR*) transcripts were found exclusively in arbuscule cells of Lotus, which suggested that the corresponding transporter may be involved in N transfer from fungus to plant before or after arbuscule disintegration. AM fungi can also take up substantial amounts of amino acids, such as glycine, glutamic acid, glutamine, and aspartic acid. The *LjLHT1.2* gene, encoding an LHT1-type amino acid transporter, was also consistently expressed in cortical cells of AM roots, where transcripts were localized mainly in arbuscular cells but also in the noncolonized cells of the root cortex [147,148,149].

### 4.4. Summary and Phylogenetic Analysis of N Transporters

**Figure 6:**
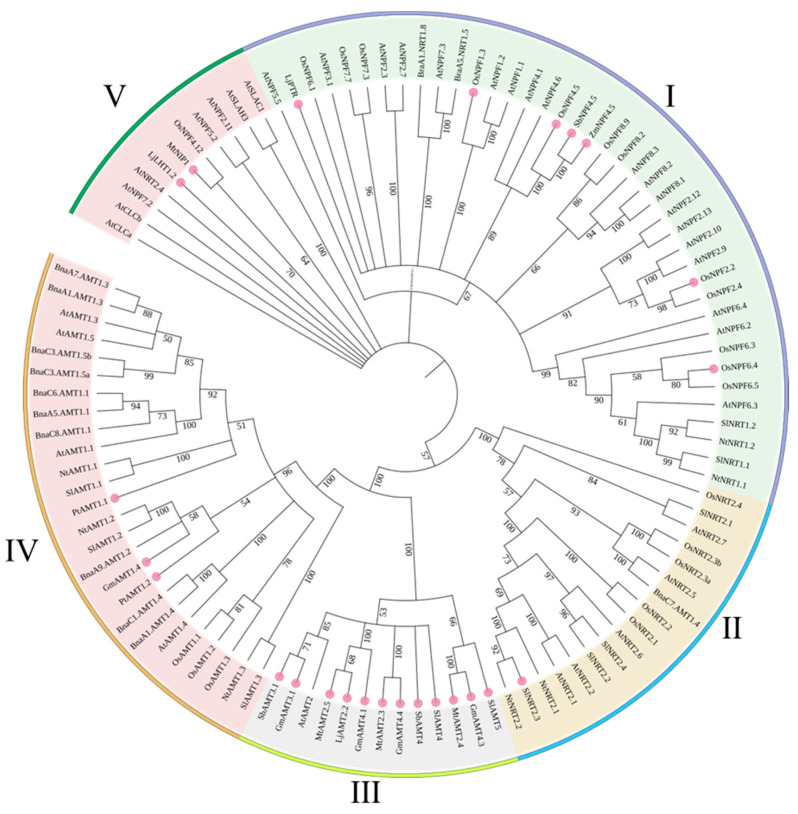
Unrooted dendrogram of plant and fungal N transporters. The unrooted phylogenetic tree of the plant nitrogen transporters homologs was constructed using their protein sequences by the neighbor joining algorithm within the MEGA7 program with bootstrapping value (range 0 to 100). For tree construction, we use nonmycorrhizal plant *Arabidopsis* and *Brassica napus* nitrogen transporters, mycorrhizal plant *Solanum Lycopersicum*, *Oryza sativa,* and *Nicotiana tabacum* nitrogen transporters, and other mycorrhizal-induced transporters (Table 2). Mycorrhizal-induced transporters are highlighted in pink color.

This coordinated and specific expression of ammonium and nitrate transporters in mycorrhizae-colonized cortical cells suggests the crucial importance of fungal N transfer in plants. Although less studied, N is also a nutritional determinant of the interaction. In this part of the review, we focus on the roles of N transporters in AM fungi and on the plant side of mycorrhizal N transporter response. We also summarize the recent advances in N uptake, assimilation and translocation in AM symbiosis, and sequence comparison between N members from the nonmycorrhizal plants *Arabidopsis* and *Brassica napus*, the mycorrhizal plants *Solanum Lycopersicum*, *Oryza sativa*, and *Nicotiana tabacum,* and other AM plant species (Figure 6). In subgroups Ⅲ, all NH_4_^+^ transporters were induced by AM except *AtAMT2*. *Arabidopsis* cannot be colonized by AM fungi, indicating that the function of AMT genes in one clade is conserved. *OsNPF4.5*, *SbNPF4.5,* and *ZmNPF4.5* were also found to cluster together, and they were strongly upregulated in roots colonized by AM fungi, suggesting that the symbiotic NO_3_^−^ uptake route might be conserved in different plant species (Figure 6). Nevertheless, this subfamily also has many other plant nitrate transporters that might be an evolutionary strategy to ensure a balance between mycorrhizal N uptake and symbiotic N fixation. Such inferences deserve further attention.

We cover the transport proteins involved in N fluxes from plants towards fungi, to the uptake from the soil and exchange of N. Interestingly, similar to P transporters, N transporters are less studied in different species of AM fungi. On the other hand, on the plant side, N transporter genes have thus far received less attention than P transporter genes in response to mycorrhizal plants. We may pay attention to N research in the future.

## 5. Regulation of Nutrient Exchange by P and N in AM Symbiosis

The availability of soil-derived nutrients has long been recognized as one of the important environmental factors controlling mycorrhizal phenotypes [150]. P and N are two major nutrient elements needed by plants. AM fungi help host plants absorb more Pi and N from the soil; in turn, Pi and N regulate AM symbiosis. Maintaining proper Pi homeostasis is important for plant growth and development, as either too-low or too-high Pi concentrations in plant cells can harm plants during symbiosis, and plants must integrate Pi status with fungal colonization and arbuscule development to maintain beneficial interactions [151]. Balzergue et al. [152] and Adeyemi et al. [153] reported that high levels of P fertilizer suppress mycorrhizal root colonization, which could be explained by the disruption of the symbiotic interaction of AM fungi by high P availability in soil. P could also inhibit AM fungi root colonization by suppressing the expression of plant symbiotic genes, especially genes encoding carotenoid and lactone biosynthesis enzymes. High levels of Pi directly inhibit spore germination by reducing the biosynthesis of strigolactone and symbiotically associated Pi transporters [153]. Plants constantly sense and signal Pi status in response to the environment. Pi starvation response 1 (PHR1) in Arabidopsis and its orthologues in other species play key roles in these processes by regulating Pi signaling and Pi homeostasis, and activate the expression of a broad range of Pi starvation-induced (PSI) genes by binding to the P1BS element in Pi-deficient conditions to improve plant Pi acquisition [154]. SPX proteins (named after the Saccharomyces cerevisiae SYG1 and Pho81 proteins and the mammalian Xpr1) have emerged as key sensors and signaling regulators of cellular Pi status in plants, and SPX proteins bind the affinity of PHRs to P1BS elements through protein–protein interactions in Pi-sufficient conditions, securing plant P homeostasis [20,151]. Recent mining of the published genomic and transcriptomic data from AM fungi detected the presence of genes encoding proteins containing the SPX domain [155]. Wang et al. [151] demonstrated that Medicago proteins SPX1 and SPX3 regulate Pi homeostasis and root colonization. Both AM symbiosis and light exposure increase nutrient uptake and utilization in plants, especially P. On the other hand, P content and the transcripts of AM-specific PTs were increased by R light under AM conditions. Light acts as a signal moving from shoots to roots in a phyB-HY5-dependent manner to regulate SL synthesis in roots. In particular, R light promotes P uptake of AM plants via both CCD7-dependent and CCD7-independent pathways [7].

It is commonly accepted that P appears to be a major regulator of AM symbiosis establishment and efficiency. In contrast, the inhibitory effect of plant N status seems to be more controversial; some studies have shown that low N stimulates mycorrhiza formation, while other studies obtained different results [156,157]. An increasing number of results suggests that N appears to be another regulator for the maintenance of the mutualistic functioning of AM symbiosis. Nouri et al. [158] reported that starvation for nitrate reversed the inhibitory effect of Pi on AM, suggesting that nutrient starvation triggers a major AM-promoting signal to counteract the effect of high Pi. Wang et al. [11] observed that, compared with 0.25 mM NO_3_^−^ conditions, rice and sorghum supplied with either 2.5 mM or 5.0 mM NO_3_^−^ results in increased AM colonization. Pan et al. [159], via gradients of long-term N addition in a Mongolian steppe, showed that low to moderate N, and N:P ratio increases were able to increase AM fungi parameters. Furthermore, Nanjareddy et al. [160] showed that NO_3_^−^ can tremendously modify mycorrhizal morphology and behavior along with plant morphology.

Recent evidence has directly demonstrated that a complex interplay occurs between N and P homeostasis [155]. P and N deficiencies had cumulative effects on AM formation; LPN (combined low P and low N) increased AM levels and significantly induced the mycorrhizal marker genes *MtPT4* and *MtBCP1* in *Medicago truncatula*. Split-root experiments further showed that AM formation in LPN plants is systematically controlled not only by P but also by N, which supports the interaction between P and N in AM symbiosis [156]. Meanwhile, downregulating PTs in *Medicago truncatula* inhibits arbuscule development, which appears to be countered by low-N conditions. This response to low N seems to involve AMT2;3, and the simultaneous mutation of this AMT and PT restores the inhibition of AM fungi colonization. This indicates that AMT-mediated N transfer to the symbiotic interface also serves as a signal to regulate mycorrhizal colonization, in some cases going beyond the function of PT [141,161,162].

## 6. Concluding Remarks and Future Perspectives

To cope with nutrient deficiency, plants have evolved several promising strategies, and AM symbiosis is one of them. To date, an increasing number of plant and fungal genes have been identified and functionally characterized that are responsible for the transport of nutrients from the soil or across the intraradical symbiotic interfaces. This review highlights some key examples to show the integrated roles of nutrient transfers in AM symbiosis. Transporters belonging to the plant or AM fungi can synergistically handle the transmembrane transport of soil nutrients to the symbiotic interface for further plant acquisition. These transporters can also promote or inhibit the colonization of AM symbiosis based on the nutrient status of plants and soil. Sequence comparison between characterized P and N transporters from the nonmycorrhizal plant Arabidopsis and *Brassica napus*, *Solanum lycopersicum*, *Oryza sativa*, *Nicotiana tabacum,* and other AM plant species illustrates that plant species capable of forming mycorrhizas possess specialized types of nutrient transporters adapted to AM symbiosis. On the other hand, in contrast to the repressed expression of the Pi transporter protein gene responsible for the direct uptake pathway, AM symbiosis tends to upregulate NO_3_^−^ transporter genes, and this difference may be partly attributed to the fact that plants require more N than P.

Although much progress has been made to elucidate the complex mechanism for the integrated roles of nutrient transfers in AM symbiosis, many questions still remain to be answered.

(1). P and N transporters are less studied in different species of AM fungi. Some mycorrhizal-specific nutrient transporter genes do not cluster with mycorrhizal-specific subgroups. It is tempting to speculate that this might be an evolutionary strategy that guarantees the balance between mycorrhizal uptake and direct uptake, and such an inference deserves further attention.
(2). The involvement of AM fungi in plant N uptake is not as clearly defined as that of P. A better understanding of the mechanism and regulation of N uptake assimilation, translocation, and transfer to the host is important for potential applications of AM fungi.
(3). Coordinated utilization of P and N is crucial for plants to maintain nutrient balance and achieve optimal growth. It has been proposed that the relative availability of soil P and N determines whether mycorrhizal benefits outweigh their costs. Increasing evidence suggests that P and N uptake and transport control mycorrhizal functioning. More studies are necessary to understand the role of mycorrhizal uptake pathways in P and N uptake.
(4). Many studies regarding transporters have been conducted on model plants, and the results have been useful in elucidating key aspects, as a next step into bringing science and agriculture. However, the symbiotic status of cultivated plants with AM fungi has not been insufficiently studied.
(5). In recent years, benefitting from the rapid progress in “omics” studies for both symbiotic partners, great progress has been made in our understanding of the P and N uptake mechanisms in AM symbiosis. Future research should address the analysis of transcriptomic and metabolomic networks at both the soil–fungi interface and fungi–plant interface to open up new perspectives in depicting a bigger picture regarding symbiosis-mediated nutrient signaling regulatory networks.

## Figures and Tables

**Table 1:** A list of phosphate transporters from different species of host plants and symbiotic fungi discussed in this article.

	Name	Accession Number	Species	References
AM fungi	GiPT	AAK72559.1	*Glomus intraradices*	[45]
	GvPT	AAC49132.1	*Glomus versiforme*	[28]
	GmosPT	AAZ22389.1	*Glomus mosseae*	[43]
	GigmPT	AHL29283.1	*Gigaspora margarita*	[47]
Plant				
Mycorrhiza-specific Pi transporters	MtPT4	AAM76744	*Medicago truncatula*	[51]
	OsPT11	AAN39052	*Oryza staiva*	[53]
	SlPT4	AAV97730	*Solanum lycopersicum*	[37]
	SlPT5	AAX85194	*Solanum lycopersicum*	[34]
	PtPT10	XP_002331845	*Populus trichocarpa*	[65]
	AsPT4	AFU50503.1	*Astralagus sinicus*	[50]
	AsPT5	AFU50504.1	*Astragalus sinicus*	[55]
	SbPT10	XP_002436966	*Sorghum bicolor*	[57]
	ZmPT6	NP_001105776	*Zea mays*	[56]
	LjPT3	BAE93353.1	*Lotus japonicus*	[54]
	GmPT10	NP_001241400	*Glysin max*	[58]
	GmPT11	AFL02621	*Glysin max*	
	StPT4	AAW51149	*Solanmum tuberosum*	[38]
	StPT5	AY885654		
	PhPt4	ACB37441		[59]
	VvPT1	XP_002267369.1	*Vitis vinifera*	[60]
	VvPT2	XP_002267327.1		
Mycorrhiza-induced Pi transporters				
	StPT3	CAC87043	*Solanmum tuberosum*	[61]
	SlPT3	AAV97729	*Solanum lycopersicum*	[34]
	SmPT3	EF091668	*Solanum melongena*	[77]
	CfPT3	ABK63962.1	*Capsicum frutescens*	
	NtPT3	EF091669	*Nicotiana tabacum*	
	ZmPT9	NP_001183901	*Zea mays*	[63]
	ZmPT1	NP_001105269		[64]
	ZmPT3	AAY42387		
	ZmPT4	AAY42388		
	ZmPT5	AAY42389		
	GmPT7	ACP19341	*Glysin max*	[58]
	PtPT8	XP_002329198	*Populus trichocarpa*	[65]
	BdPT7	XP_010229243	*Brachypodium distachyon*	[66]
	BdPT12	XP_003581013		
	BdPT13	XP_003581014		
	BdPT3	XP 003557302.1		
	OsPT13	AAN39054	*Oryza satival*	[62]
	LjPT4	BAG71408	*Lotus japonicus*	[69]
	HvPHT1;11	XP_044983919	*Hordeum vulgare subsp. vulgare*	[67]
	HvPHT1;12	XP_044953977		
	HvPHT1;13.1	XP_044969167		
	HvPHT1;13.2	XP_044969168		
	HvPT8	AY187023		[68]
	TaPht-myc	AJ830009	*Triticum aestivum*	[68]
	SbPT9	EES10479	*Sorghum bicolor*	[78]
	SbPT8	XP_002464558		
	SbPT11	XP_002458253		
	AsPT1	AFU50500.1	*Astralagus sinicus*	[50]
	LuPT5	Lus10014754	*Linum usitatissimum*	[78]
	LuPT8	Lus10012860		
	PhPT3	ACB37440	*Petunia hybrida*	[59]
	PhPT5	ACB37442		
H^+^-ATPase				
	OsHA1	BAS81814	*Oryza staiva*	[71]
	MtHA1	CAB85494	*Medicago truncatula*	[70]
	SlHA8	Solyc08g078200.2.1	*Solanum lycopersicum*	[73]
	PMA2	4DX0_A	*Nicotiana tabacum*	[72]
	PMA4	3M51_A		
Downregulated Pi transporters	OsPT1	XP_015631295	*Oryza satival*	[75]
	OsPT2	XP_015630484		
	OsPT3	XP_015614123		
	OsPT6	XP_015649112		
	OsPT9	AAN39050		
	OsPT10	AAN39051		
	StPT1	NP_001275200	*Solanum tuberosum*	[61]
	StPT2	CAA67396		

**Table 2:** A list of nitrogen transporters from different species of host plants and symbiotic fungi discussed in this article.

Nutrients	Accession Number	Name	Species	References
Nitrogen				
AM fungi				
Ammonium	CAI54276	GintAMT1	*Rhizophagus irregularis*	[131]
	CAX32490	GintAMT2		[129]
	ANI87614	GintAMT3		[125]
Nitrate	XP_658612.1	GiNT		[128]
Organic nitrogen	AAX81451	GmosAAP1	*Funneliformis mosseae*	[135]
	XP_025186378	RiPTR2	*Rhizophagus irregularis*	[136]
Plant				
Ammonium	XP_025979915	GmAMT1.4	*Glysin max*	[138]
	XP_003524319	GmAMT3.1		
	XP_003533686	GmAMT4.1		
	XP_003553758	GmAMT4.3		
	XP_014626736	GmAMT4.4		
	ACQ91094	LjAMT2.2	*Lotus japonicus*	[137]
	XP_002311703	PtAMT1.1	*Populus trichocarpa*	[139]
	XP_024439713	PtAMT1.2		
	XP_002456706	SbAMT3.1	*Sorghum bicolor*	[140]
	XP_021307349	SbAMT4		
	XM_019215621.2	SlAMT4	*Solanum lycopersicum*	[115]
	XM004245353.2	SlAMT5		
	XP_003629223	MtAMT2.3	*Medicago truncatula*	[141]
	G7L1W7	MtAMT2.4		
	A0A072VHJ1	MtAMT2.5		
	AAL32128	MtNIP1		[142]
Nitrate				
	NP_001234127	SlNRT2.3	*Solanum lycopersicum*	[105]
	XP_015621687	OsNPF4.5	*Oryza sativa*	[11]
	XP_020406064.1	ZmNPF4.5	*Zea mays*	
	XP_021311980.1	SbNPF4.5	*Sorghum bicolor*	
	XP_015620477.1	OsNPF2.2	*Oryza sativa*	[145]
	XP_015636060.1	OsNPF1.3		
	XP_015632236.1	OsNPF6.4		
Organic nitrogen	AAB69642	LjPTR	*Lotus japonicus*	[146]
	AEE98384	LjLHT1.2	*Lotus japonicus*	[147]

## Data Availability

Not applicable.
